# Assessment of antimicrobial prescribing patterns, guidelines compliance, and appropriateness of antimicrobial prescribing in surgical-practice units: point prevalence survey in Malaysian teaching hospitals

**DOI:** 10.3389/fphar.2024.1381843

**Published:** 2024-04-24

**Authors:** Nurul Adilla Hayat Jamaluddin, Petrick Periyasamy, Chee Lan Lau, Sasheela Ponnampalavanar, Pauline Siew Mei Lai, Ly Sia Loong, Tg Mohd Ikhwan Tg Abu Bakar Sidik, Ramliza Ramli, Toh Leong Tan, Najma Kori, Mei Kuen Yin, Nur Jannah Azman, Rodney James, Karin Thursky, Isa Naina Mohamed

**Affiliations:** ^1^ Pharmacoepidemiology and Drug Safety Unit, Department of Pharmacology, Faculty of Medicine, Universiti Kebangsaan Malaysia, Kuala Lumpur, Malaysia; ^2^ Department of Hospital and Clinical Pharmacy, Faculty of Pharmacy, University of Cyberjaya, Cyberjaya, Selangor, Malaysia; ^3^ Medical Department, Faculty of Medicine, Universiti Kebangsaan Malaysia, Kuala Lumpur, Malaysia; ^4^ Pharmacy Department, Hospital Canselor Tuanku Muhriz, Kuala Lumpur, Malaysia; ^5^ Department of Medicine, Faculty of Medicine, University of Malaya, Kuala Lumpur, Malaysia; ^6^ Department of Primary Care Medicine, Faculty of Medicine, University of Malaya, Kuala Lumpur, Malaysia; ^7^ School of Medical and Life Sciences, Sunway University, Petaling Jaya, Selangor, Malaysia; ^8^ Department of Medical Microbiology and Immunology, Faculty of Medicine, Universiti Kebangsaan Malaysia, Kuala Lumpur, Malaysia; ^9^ Emergency Department, Faculty of Medicine, Universiti Kebangsaan Malaysia, Kuala Lumpur, Malaysia; ^10^ The Royal Melbourne Hospital, Melbourne, Australia; ^11^ National Centre for Antimicrobial Stewardship, Department of Infectious Diseases, University of Melbourne, Melbourne, Australia

**Keywords:** point prevalence, guidelines compliance, appropriateness, surgical, antimicrobial prophylaxis, antimicrobial stewardship

## Abstract

**Objectives:** This study sought to investigate the quality of antimicrobial prescribing among adult surgical inpatients besides exploring the determinants of non-compliance and inappropriate prescribing to inform stewardship activities.

**Methods:** A cross-sectional point prevalence study employing Hospital National Antimicrobial Prescribing Survey (Hospital NAPS) was conducted in April 2019 at two teaching hospitals in Malaysia.

**Results:** Among 566 surgical inpatients, 44.2% were receiving at least one antimicrobial, for a total of 339 prescriptions. Antimicrobials belonging to the World Health Organization’s Watch group were observed in 57.8% of cases. Both hospitals exhibited similar types of antimicrobial treatments prescribed and administration routes. A significant difference in antimicrobial choice was observed between hospitals (*p* < 0.001). Hospital with electronic prescribing demonstrated better documentation practice (*p* < 0.001). Guidelines compliance, 32.8% (*p* = 0.952) and appropriateness, 55.2% (*p* = 0.561) did not significantly differ. The major contributors of inappropriateness were incorrect duration, (15%) and unnecessary broad-spectrum coverage, (15.6%). Non-compliance and inappropriate prescribing were found to be 2 to 4 times significantly higher with antimicrobial prophylaxis prescription compared to empirical therapy.

**Conclusion:** Antimicrobial stewardship efforts to improve appropriate surgical prescribing are essential. These initiatives should prioritize surgical prophylaxis prescribing, focusing on reducing unnecessarily prolonged use and broad-spectrum antimicrobials, raising awareness among prescribers and promoting proper documentation.

## 1 Introduction

Rapid development of antimicrobial resistance (AMR) has become a serious healthcare issue in recent decades (Rice, 2009; Murray et al., 2022). Unchecked use of antimicrobials resulting in their overuse and misuse is driving the acceleration of this issue, which has a direct impact on the healthcare system (Laxminarayan et al., 2013; Allcock et al., 2017). Hence, identifying and stopping inappropriate antimicrobial prescribing is essential to slow the emergence and spread of AMR organisms. In response to the World Health Organization (WHO) Global Action Plan to combat AMR, Malaysia has formulated the Malaysian Action Plan on Antimicrobial Resistance (MyAP-AMR), under a One Health approach targeting to reduce inappropriate antimicrobial use in human and animal health (Ministry of Health Malaysia, 2017; Ministry of Health Malaysia, 2022a). Similarly, the United States’ National Strategy for Combating Antibiotic-Resistant Bacteria (CARB) include a target of reducing inappropriate prescribing by 20% in hospital settings (National Action Plan for combating antibiotic-resistant bacteria, 2015). In line with these goals, point prevalence surveys (PPS) of antimicrobial utilization and audit on compliance with national or local guidelines were integrated into the antimicrobial stewardship (AMS) program as part of this national strategy (Ministry of Health Malaysia, 2022b).

A 3-year observation study in a Malaysian hospital from 2018 to 2020 identified a concerning correlation between the increased consumption of broad-spectrum antibiotics and the rise of multidrug resistant organisms, underscoring the urgency of addressing this growing trend in Malaysia (Tan et al., 2022). Although resistance patterns of certain pathogens such as *Staphylococcus aureus* and *Streptococcus pneumoniae* remained stable over 5-year period, *Methicillin-resistant Staphyloccocus aureus* (MRSA) showed a downward trend. Conversely, *Acinetobacter baumannii* demonstrated a worrisome increase in resistance to various antibiotics, with rates as high as 68.8% for imipenem and meropenem in 2021. Similarly, *Pseudomonas aeruginosa* exhibited an upward trend in resistance, while *Klebsiella pneumonia* and *Escherichia coli* displayed a doubling in resistance to carbapenems over the same period (Ministry of Health Malaysia, 2022a).

Furthermore, antimicrobial consumption rates in this country remain high despite efforts to curb their discriminate use. Total antibiotic utilization has shown an upward trend in all areas, particularly in intensive care units (ICUs), suggesting the need for targeted interventions in hospital settings (Ministry of Health Malaysia, 2022a; Pharmacy Practice and Development Division and Ministry of Health, 2022). In 2018, while low- and middle-income countries (LMICs) recorded an antibiotic consumption rate of 13.1 DDD per population 1,000 per day, Malaysia reported a lower rate of 9.9 DDD per 1,000 per day, ranking behind Vietnam (30 DDD per 1,000 per day) and Thailand (12.4 DDD per 1,000 per day) (Browne et al., 2021). Comparatively, the country demonstrated a concerning high antibiotic usage of 79%, surpassing larger neighboring countries such as Philippines (42%) and Indonesia (43%) (Browne et al., 2021).

In surgical practice, antimicrobials are used widely for both prophylactic and medical treatment. Evidence-based national and local antimicrobial guidelines for surgical practices, including surgical prophylactic use have been published and constantly updated. Despite evidence suggesting that good practice is sufficient, hospitals are still struggling to comply (Gul et al., 2005; Ng and Chong, 2012; Oh et al., 2014; Lim et al., 2015; Bardia et al., 2021; Cabral et al., 2023). While studies have assessed the appropriateness of antimicrobial prescribing across various specialties (Charani et al., 2019; Sheng et al., 2019; Vandael et al., 2020; de Guzman Betito et al., 2021; Macera et al., 2021), it is important to recognize that the conditions for which antimicrobials are prescribed can differ in surgical practices, even though the principles of infection diagnosis and management remain the same. Data from National Antibiotic Utilisation survey in 2015 and 2016 revealed that only a small percentage of in-patient prescriptions (5.7%) were for surgical prophylaxis, 2.6% for non-surgical prophylaxis, and the remaining majority were for therapeutic indications (Ministry of Health Malaysia, 2020). A study in surgical wards found 86% of antibiotics were prescribed for therapeutics, and highlighted significant inappropriate prescribing practices in the wards, indicating a need for improved compliance with guidelines (Lim et al., 2015). Notably, most literature on antimicrobial prescribing in surgical practices in the country focuses on surgical antimicrobial prophylaxis (SAP) (Gul et al., 2005; Oh et al., 2014; Fadzwani et al., 2020; Zammari et al., 2022), leaving the gap in understanding broader antimicrobial prescribing patterns in surgical units.

To assess antimicrobial use and prescribing quality, the Royal Melbourne Hospital developed the Hospital National Antimicrobial Prescribing Survey (Hospital NAPS) (National Centre for Antimicrobial Stewardship, 2023), a validated web-based auditing platform, delivered by the National Centre for Antimicrobial Stewardship (NCAS) in collaboration with the Australian Government Department of Health and Aged Care, to monitor the performance of AMS program in hospitals. The platform enables multidisciplinary healthcare professionals across various healthcare institutions to identify focus areas and benchmark the performance indicators among participating hospitals in a standardized manner. The anonymized aggregate survey data from Hospital NAPS has facilitated the establishment of the Antimicrobial Use and Resistance in Australia (AURA) surveillance system, which informs national AMS strategies and assists in the regular review and updating of prescribing guidelines (Australian Commission on Safety and Quality in Health, 2021). Since its successful implementation in Australia, Hospital NAPS has been adopted by other countries with varied healthcare systems, including Canada and Bhutan; demonstrating the feasible, generalizable, with potential to optimize antimicrobial use (James et al., 2022).

Limited information regarding antimicrobial prescribing for different infection diagnoses in surgical settings suggesting a clear need for more comprehensive data in these contexts to guide tailored AMS initiatives and approaches. Such knowledge is vital to shift from a one-size-fits-all model to one that addresses the specific challenges faced by prescribers in surgical units. Using the Hospital NAPS protocol, this study sets out to investigate and report an in-depth picture of antimicrobial prescribing patterns among surgical inpatients and evaluates the prescribing quality in surgical-practice units in two teaching hospitals in Malaysia, including compliance with guidelines and reasons for inappropriate prescribing. The findings from this study can facilitate comparative studies with other surgical populations, and inform more specific investigations.

## 2 Materials and methods

### 2.1 Study design and settings

A hospital-wide cross-sectional point prevalence survey (PPS) of antimicrobial prescribing was performed in two teaching hospitals in Klang Valley, Malaysia (Jamaluddin et al., 2021). Hospital Canselor Tuanku Muhriz or HCTM (1,054 beds, 63 wards) and University Malaya Medical Centre or UMMC (1,617 beds, 44 wards) are university-affiliated hospitals with multidisciplinary AMS teams. PPS was conducted for each facility on designated days between 16 April to 30 April 2019. Auditors were assigned a specific day to complete a standard Hospital NAPS protocol, completing a data collection form for each patient prescribed with an antimicrobial on the designated audit day (Supplementary Material). A detailed description of the Hospital NAPS antimicrobial prescribing surveys is described in previous publications (James et al., 2014; James et al., 2022). Survey and assessment were executed by fourteen pharmacists and two infectious disease (ID) physicians in HCTM, while one pharmacist and four ID physicians undertook the exercises in UMMC. The Australian NAPS support team provided training, technical and clinical support throughout the survey period. All surveyors received online webinar training on the audit protocol before the survey day. Data collected during the survey were compiled and submitted through a secure web-based online platform. Data on antimicrobial prescribing among patients admitted to surgical-practice units were analyzed for this report. The study was approved and ethics approval from each institution was obtained before the commencement of this study.

### 2.2 Eligibility criteria/patient selection

All adult patients admitted to the obstetrics and gynecology (OBGYN), trauma and orthopedic and surgical specialties, before or at 8 a.m. on the day of the survey were audited once (denominator). Patients admitted after 8 a.m., outpatients, as well as patients undergoing same-day treatment and surgery in daycare or at emergency unit, were excluded. The following information were retrieved from medical records and associated documents for patients who were prescribed with at least an antimicrobial (numerator) regardless of route of administration: demographics, diagnosis, antimicrobial data (including indications, dose, route, frequency, duration, start and review/stop date) and any additional clinical variables (cultures, biomarkers) relevant for the assessment. The survey also included patients who were prescribed a stat dose of antimicrobial or SAP since 8 a.m. the previous day. A unique, non-identifiable survey number was assigned to every de-identified patient data. Aligning with established protocols by Hospital NAPS (James et al., 2014; James et al., 2022) and WHO (World Health Organization, 2018), setting 8 a.m. as the cut-off time for patient inclusion ensures comprehensive representation of all admitted patients while minimizing variability of different time points and the capture of diverse sample encompassing individuals who have undergone consultations and received treatment. Additionally, corresponding with the facilities’ operational day, this timing facilitates efficient data collection by the survey team. This method strikes a balance between practical considerations and the imperative to obtain a representative patient group, plus ensuring consistency and comparability with existing literature. The calculated minimum sample size, determined by the Krejcie and Morgan formula, was 256 subjects. This estimation was based on a preliminary survey conducted in the hospital, which reported a prevalence of antimicrobial use at 78.9%, considering type 1 error rate of 5% and a precision of 5% (Krejcie and Morgan, 1970).

### 2.3 Assessment

#### 2.3.1 Compliance with guidelines

To meet “guideline compliant” assessment criteria, the prescription must be the first-line or preferred recommendations outlined in the primary guidelines. Doses were also evaluated using the hospital renal dose adjustment protocol, if necessary. HCTM followed the Malaysian National Antibiotic Guideline 2014 (Ministry of Health, 2014) and the hospital surgical prophylaxis guide as the main prescribing guidelines; while UMMC adhered to the UMMC antibiotic guideline (University Malaya Medical Center, 2020) available at the time of assessment. The evaluation was based on the information documented in the patient records. When clear recommendations were lacking in the primary references, a consensus was reached among the experts; including ID physicians and clinical pharmacists. The consensus was achieved either with or without consulting additional sources, such as international guidelines or ward protocols. Categories in accordance to the Hospital NAPS were compliant, non-compliant, directed therapy (prescribing guided by microbiology and susceptibility results), non-assessable due to insufficient reports or unclear diagnosis, or no guidelines available.

#### 2.3.2 Appropriateness

The Hospital NAPS defines appropriateness as the degree to which antimicrobial prescribing aligns with the primary references or best practices endorsed by experts (optimal); or considered reasonable alternative (adequate). Prescriptions that deviate from these standards are deemed inappropriate, either suboptimal or inadequate. Suboptimal prescribing encompasses prescription where antimicrobial choice is unreasonably broad in spectrum, dosage is excessively high, or duration is prolonged, including failure to de-escalate empirical to targeted therapy. This category also includes cases where the prescribed antimicrobial does not match the patient’s allergy profile, potentially resulting in mild adverse reactions. Inadequate prescriptions are those unlikely to effectively treat the infection, or unnecessary for the given indication. These prescriptions may pose severe or life-threatening toxicity risks, or when SAP is unnecessarily prolonged beyond 24 h (Supplementary Material).

### 2.4 Data analysis

Antibiotics were classified as “Access,” “Watch” and “Reserve” (AWaRe) according to the 2021 WHO AWaRe classification (World Health Organization, 2021). Antimicrobials not included in the AWaRe classification were listed as “unclassified.” Details on AWaRe classification for the type of treatment are shown in Supplementary Material. Continuous data were presented as the mean and standard deviation (SD) for normally distributed data. If the distribution was not normal, continuous data were presented as the median and interquartile range (IQR). Other descriptive statistics, such as minimum and maximum values were reported when necessary. Normality of the data was examined using histogram (approximately bell-shaped), skewness (within −1 to 1) and kurtosis (within −3 to 3). The difference between hospitals was analyzed using the Chi-square test or Fisher’s exact test (if minimum expected count was less than 5) for categorical variables. For continuous age variables, independent *t*-test was used to analyze the mean difference between hospitals. Compliance with guidelines and appropriateness were treated as dichotomous variables. The associations of each potential factor with compliance and appropriateness were examined through the Chi-square test or Fisher’s exact test. Multiple logistic regressions were used to evaluate significant factors. Odds ratio and 95% confidence interval for each potential factor were calculated, where a *p*-value of less than 0.05 was considered significant. All analyses were carried out using SPSS (IBM Corp. released 2011 IBM SPSS Statistics for Windows, Version 22.0. Armonk, NY: IBM Corp).

## 3 Results

### 3.1 Demographics and prevalence

A total of 229 admissions in HCTM from twenty wards plus one burn unit, and 337 in UMMC from thirteen wards were identified. Admissions to the surgical and burn units accounted for 51.1% (289) of patients, followed by OBGYN with 24.4% (138), trauma and orthopedic with 22.6% (128) and mix ward with 1.9% (Browne et al., 2021). Among 566 patients, 250 (44.2%) received at least one antimicrobial prescription at the time of the survey, for a total of 339 prescriptions (median 1 per patient, range 1–5), with 171 (68.4%) receiving one antimicrobial agent, 71 (28.4%) receiving two and 8 (3.2%) receiving three or more. Demographic data is presented in Table 1.

**TABLE 1:** Total admissions (*n* = 566) and the general characteristics of patients on antimicrobials in surgical wards (*n* = 250).

Characteristics, n	Total	HCTM, *n* (%)	UMMC, *n* (%)	*p*-value^a^
No. of surgical patients	566	229	337	
No. of patients on antimicrobials, *n* (%)	250 (44.2)	88 (38.4)	162 (48.1)	0.023
Surgical-practice specialties, *n* (%)				
General surgery^d^	68 (27.2)	27 (30.7)	41 (25.3)	0.217^c^
Cardiothoracic	12 (4.8)	3 (3.4)	9 (5.6)	
Neurosurgery	16 (6.4)	4 (4.5)	12 (7.4)	
Urology	22 (8.8)	9 (10.2)	13 (8)	
Ophthalmology	6 (2.4)	5 (5.7)	1 (0.6)	
OBGYN	33 (13.2)	8 (9.1)	25 (15.4)	
Trauma and orthopedic	84 (33.6)	29 (33)	55 (34)	
Others^e^	9 (3.6)	3 (3.4)	6 (3.7)	
Mean (SD) age of patients (years)		56.06 (18.17)	54.80 (18.18)	0.601^b^
Age Group, *n* (%)				
<30 years	29 (11.6)	10 (11)	19 (12)	0.717
30–49 years	58 (23.2)	16 (18)	42 (26)	
50–64 years	73 (29.2)	28 (32)	45 (28)	
65–79 years	75 (30.0)	28 (32)	47 (29)	
≥80 years	15 (6.0)	6 (7)	9 (6)	
Gender, *n* (%)				
Male	130 (52)	52 (59)	78 (48)	0.098
No. of prescriptions per patient, *n* (%)				
1	171 (68.4)	64 (72.7)	107 (66)	0.081^c^
2	71 (28.4)	19 (21.6)	52 (32.1)	
≥3	8 (3.2)	5 (5.7)	3 (1.9)	

^a^ Chi-squared test.

^b^ Independent *t*-test.

^c^ Fisher Exact test.

^d^ General surgery: inclusive of general surgery, breast and endocrine surgery, colorectal surgery, gastrointestinal and bariatric, hepatobiliary and pancreatic, and vascular surgery.

^e^ Others: inclusive of plastic surgery, oral and maxillofacial surgery, and ENT.

HCTM, Hospital Canselor Tuanku Muhriz; UMMC, University Malaya Medical Centre; OBGYN, obstetrics and gynecology.

### 3.2 Antimicrobial prescribing patterns

Common types of treatment and route of antimicrobials administration were seen to be prescribed in both hospitals (*p* > 0.05), but UMMC demonstrated better rates (>95%) for documentation practice (*p* < 0.001) (Table 2). Of all agents prescribed empirically, 51.2% (86/168) were in the Watch group [piperacillin/tazobactam (31.4%) and cefuroxime (29%)], while Access antibiotics accounted for 42% (74/168) of prescriptions. More than half (59.6%; 62/104) of all antimicrobials prescribed prophylactically were Watch antibiotics constituted mainly by cefuroxime (54.8%). Directed therapy was largely entailing antibiotics of Watch by 71.6% (48/67), where meropenem (19%, 13), cefepime (13%, 9) and vancomycin (13%, 9) were prescribed. Access antibiotics were higher in HCTM (49.6%, 59), while the use of Watch antibiotics was found to be higher in UMMC (64.1%, 141) (*p* = 0.005).

**TABLE 2:** Antimicrobial prescription details (*n* = 339).

Characteristics	Total	HCTM, *n* (%)	UMMC, *n* (%)	*p*-value^a^
Number of prescriptions	339	119	220	
Type of treatment				
Empiric	168 (49.6)	64 (53.8)	104 (47.3)	0.179
Directed therapy	67 (19.8)	26 (21.8)	41 (18.6)	
Prophylaxis	104 (30.7)	29 (24.4)	75 (34.1)	
Route of administration				
Intravenous	252 (74.3)	86 (72.3)	166 (75.5)	0.039
Oral/enteral	63 (18.6)	19 (16.0)	44 (20.0)	
Others^c^	24 (7.1)	14 (11.8)	10 (4.5)	
Reason for antimicrobials documented				
Yes	292 (86.1)	81 (68.1)	211 (95.9)	<0.001
No	47 (13.9)	38 (31.9)	9 (4.1)	
Stop/review date documented				
Yes	241 (71.1)	26 (21.8)	215 (97.7)	<0.001
No	98 (28.9)	93 (78.2)	5 (2.3)	
AWaRe category				
Access prescription	133 (39.2)	59 (49.6)	74 (33.6)	0.005^b^
Watch prescription	196 (57.8)	55 (46.2)	141 (64.1)	
Reserve prescription	1 (0.3)	1 (0.8)	0	
Unclassified	9 (2.7)	4 (3.4)	5 (2.3)	
Antimicrobial pharmacological group				
Penicillin	112 (33.0)	60 (50.4)	52 (23.6)	<0.001
Cephalosporin	104 (30.7)	23 (19.3)	81 (36.8)	
Nitroimidazole	34 (10.0)	7 (5.9)	27 (12.3)	
Carbapenem	20 (5.9)	2 (1.7)	18 (8.2)	
Quinolone	17 (5.0)	11 (9.2)	6 (2.7)	
Others^d^	52 (15.3)	16 (13.4)	36 (16.4)	
Compliance with guideline^e^				
Compliance	83 (32.8)	28 (32.6)	55 (32.9)	0.952
Non-compliance	170 (67.2)	58 (67.4)	112 (67.1)	
Appropriateness^f^				
Appropriate (optimal, adequate)	180 (55.2)	61 (53)	119 (56.4)	0.561
Inappropriate (suboptimal, inadequate)	146 (44.8)	54 (47.0)	92 (43.6)	

^a^ Chi-squared test.

^b^ Fisher-Exact test.

^c^ Others: inclusive of vaginal, inhalation and topical routes.

^d^ Others: inclusive of aminoglycosides, amphenicol, carboxylic acid, Fusidane, Glycopeptide, Lincomycin, Macrolide, Nitrofuran, Sulfonamide, antituberculosis, antifungal.

^e^ Exclude directed therapy, no guidelines available for the specific indication, and not assessable compliance, *n* = 253.

^f^ Exclude prescriptions with no guidelines available for the specific indication, and not assessable appropriateness, *n* = 326.

HCTM, Hospital Canselor Tuanku Muhriz; UMMC, University Malaya Medical Centre; OBGYN, obstetrics and gynecology.

Antimicrobial were mostly prescribed for surgical prophylaxis (27.1%, 92), followed by cystitis (4.7%, 16), necrotizing fasciitis (4.4%, 15) and acute cholecystitis (4.1%, 14). There was a significant difference in the choice of antimicrobial between hospitals (*p* < 0.001). Cefuroxime (25.5%, 56) and metronidazole (12.3%, 27) were the most commonly used antimicrobials at UMMC, while HCTM recorded the most frequent use of amoxicillin/clavulanic acid (23.5%, 28). From 92 antimicrobial prescriptions for surgical prophylaxis, cephalosporins (53.3%, 49) accounted for predominant choices. The five most used SAP in both hospitals were cefuroxime (37%, 34), metronidazole (18.5%, 17), amoxicillin/clavulanic acid (12.0%, 11), ceftriaxone (7.6%, 7) and vancomycin (5.4%, 5). UMMC mainly utilized cefuroxime (48.5%, 33/68), metronidazole (23.5%, 16/68) and vancomycin (7.4%, 5/68), while HCTM’s preferred choice was amoxicillin/clavulanic acid (41.7%, 10/24). A remarkable use of ceftriaxone (29.2%, 7/24) for SAP in HCTM was observed.

### 3.3 Compliance with guidelines and appropriateness

The study revealed a compliance rate with guidelines was at 32.8% and an appropriateness level at 55.2%. Both indicators displayed no statistically significant difference between the two hospitals (Table 2). Of 146 (44.8%) prescriptions that were assessed as inappropriate, 72 (22.1%) were classified as suboptimal while the remaining 74 (22.7%) were classified as inadequate. The percentage of prescriptions judged suboptimal and inadequate did not differ between hospitals with *p* = 0.219 and *p* = 0.056, respectively. Inappropriate prescribing varied by subspecialties, overall ranging from 40.9% to 58.3%. A group of units inclusive of plastic surgery, oral and maxillofacial surgery and ENT (others) had the highest percentage of inappropriate orders at 58.3% (7/12), along with cardiothoracic at 57.1% (8/14), ophthalmology at 52.9% (9/17) and OBGYN at 46.9% (23/49). HCTM recorded inappropriateness ranging from 30.8% to 100%, with high rates in cardiothoracic, neurosurgery and others. Meanwhile, the tabulation in UMMC revealed ophthalmology, urology and others as among the units with a high percentage of inappropriate orders ranging from 35.3% to 100%.

Prophylaxis (medical and surgical) prescriptions had the highest inappropriateness (*n* = 69/146, 47.3%) compared to empirical and directional therapy. The greatest percentage of inappropriate prescriptions was SAP with 40 (43.5%) of 92 prescriptions classified as inadequate and 27 (29.3%) as suboptimal. Both hospitals recorded a high number of inappropriate SAP orders presenting 83% (20/24) in HCTM and 69% (47/68) in UMMC. Unnecessary prolongation ≥24 h was the most common reason for inappropriate prescribing of SAP prescriptions, respectively; 50% (12/24) in HCTM and 38.2% (26/68) in UMMC.

**FIGURE 1:**
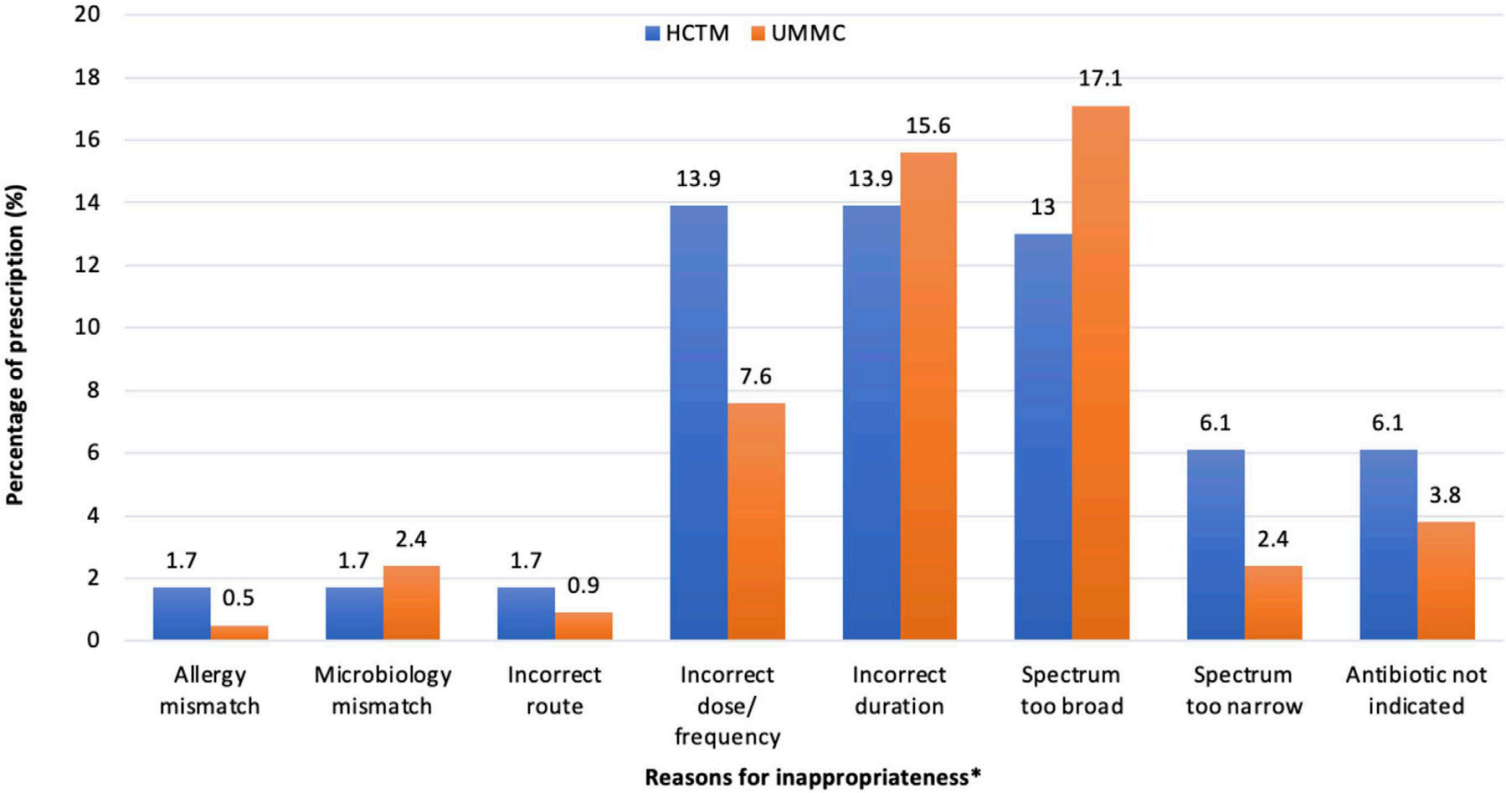
Reasons for a prescription being assessed as inappropriateness in HCTM and UMMC (n = 146). *A prescription may have more than one reason of inappropriateness. Spectrum too broad: Antimicrobials that have a spectrum of activity that exceeds the requirements for the specific clinical indication, as outlined by the recommended guidelines or microbiological susceptibility results. This may include prescribing broad-spectrum antimicrobial without de-escalating to a narrower spectrum based on microbiological results or prescribing multiple antimicrobials with unnecessary overlap in spectrum. Spectrum too narrow: Antimicrobials that do not adequately cover the likely causative or cultured pathogens for the given condition.

A sub-analysis of 146 inappropriate prescriptions is shown in Figure 1. Total rates of SAP ≥24 h (41.3%, 38/92) contributed mainly to the incorrect duration of antimicrobials in overall prescriptions (15%, 49/326). The extensive use of broad-spectrum antimicrobials in the overall prescribing was depicted at 15.6% (51/326). A higher rate of a broader spectrum of antimicrobials was noted in UMMC (17.1%, 36/211), while incorrect dosage/frequency (13.9%, 16/115) was more commonly seen in HCTM.

### 3.4 Factors associated with non-compliance and inappropriateness

The results of univariate and multivariate models for both hospitals are presented in Tables 3, 4. Non-compliance and inappropriate antimicrobial prescriptions were more frequently associated with prophylaxis indications compared to empirical and directed therapy. The likelihood of antimicrobial prophylaxis prescriptions being non-compliant was 4.5 times higher (OR 4.55, 95% CI 1.40–14.78, *p* = 0.012), and 4.2 times more likely to be found as deemed inappropriate (OR 4.22, 95% CI 1.61–11.10, *p* = 0.003) in HCTM. Conversely, UMMC showed 2.4 times (OR 2.37, 95% CI 1.21–4.65, *p* = 0.012) higher likelihood of inappropriateness in prescribing antimicrobial prophylaxis. General surgery (OR 12.56, 95% CI 1.82–86.48, *p* = 0.010), OBGYN (OR 29.89, 95% CI 3.78–236.49, *p* = 0.001) as well as trauma and orthopedic (OR 8.06, 95% CI 1.25–52.11, *p* = 0.028) had significantly higher odds of non-compliance with guidelines compared to cardiothoracic unit. Additionally, prescribing cephalosporins was significantly associated with higher likelihood of non-compliance with guidelines (OR 8.57, 95% CI 2.89–25.39, *p* < 0.001) compared to penicillins.

**TABLE 3:** Factors associated with non-compliance with guidelines in HCTM and UMMC.

	HCTM					UMMC				
Characteristics	Non-compliance (*n* = 58)	Compliance (*n* = 28)	*p*-value^a^	Crude odd ratio (95% CI)	^c^ *p*-value^a^	Non-compliance (*n* = 112)	Compliance (*n* = 55)	*p*-value^a^	Adjusted odd ratio (95% CI)	^c^ *p*-value^a^
Type of treatment, *n* (%)					0.012					0.076
Empiric	33 (57.9)	24 (42.1)	0.014	1.00 (Reference)		53 (57.0)	40 (43.0)	0.003	1.00 (Reference)	
Prophylaxis	25 (86.2)	4 (13.8)		4.55 (1.40–14.78)	0.012	59 (79.7)	15 (20.3)		2.43 (0.91–6.47)	0.076
Subspecialties Group, *n* (%)										0.043
Cardiothoracic	2 (100.0)	0 (0.0)	0.198^b^			3 (33.3)	6 (66.7)	0.001^b^	1.00 (Reference)	
General surgery	13 (54.2)	11 (45.8)				31 (70.5)	13 (29.5)		12.56 (1.82–86.48)	0.010
Neurosurgery	2 (40.0)	3 (60.0)				4 (36.4)	7 (63.6)		5.15 (0.47–56.16)	0.179
OBGYN	9 (90.0)	1 (10.0)				30 (88.2)	4 (11.8)		29.89 (3.78–236.49)	0.001
Ophthalmology^d^	7 (53.8)	6 (46.2)				1 (100.0)	0 (0.0)		NA	NA
Trauma and orthopedic	18 (75.0)	6 (25.0)				30 (60.0)	20 (40.0)		8.06 (1.25–52.11)	0.028
Urology	5 (83.3)	1 (16.7)				7 (58.3)	5 (41.7)		7.92 (0.88–70.89)	0.064
Others^d^	2 (100.0)	0 (0.0)				6 (100.0)	0 (0.0)		NA	NA
Antimicrobial group, *n* (%)										< 0.001
Penicillin	32 (66.7)	16 (33.3)	0.234^b^			17 (40.5)	25 (59.5)	< 0.001^b^	1.00 (Reference)	
Cephalosporin	12 (80.0)	3 (20.0)				62 (88.6)	8 (11.4)		8.57 (2.89–25.39)	< 0.001
Carbapenem	0 (0.0)	1 (100.0)				3 (75.0)	1 (25.0)		10.56 (0.65–171.44)	0.097
Nitroimidazole	2 (33.3)	4 (66.7)				21 (84.0)	4 (16.0)		2.78 (0.68–11.41)	0.155
Quinolone	7 (70.0)	3 (30.0)				2 (40.0)	3 (60.0)		0.64 (0.05–8.04)	0.731
Other	5 (83.3)	1 (16.7)				7 (33.3)	14 (66.7)		0.65 (0.19–2.25)	0.493

^a^ Chi-Squared test.

^b^ Fisher Exact test. Odd ratio based on non-compliance group (non-compliance/compliance).

^c^ *p*-value for Adjusted Odd Ratio.

^d^ Excluded in the multivariate analysis due to small number.

HCTM, Hospital Canselor Tuanku Muhriz; UMMC, University Malaya Medical Centre; OBGYN, obstetrics and gynecology.

**TABLE 4:** Factors associated with inappropriate prescribing in HCTM and UMMC.

Hospital	HCTM					UMMC				
Characteristics	Inappropriate (*n* = 54)	Appropriate (*n* = 61)	*p*-value^a^	Crude odd ratio (95% CI)	^c^p-value^a^	Inappropriate (*n* = 92)	Appropriate (*n* = 119)	*p*-value^a^	Adjusted odd ratio (95% CI)	^c^p-value^a^
Type of treatment, *n* (%)					< 0.001					< 0.001
Empiric treatment	23 (38.3)	37 (61.7)	0.006	1.00 (Reference)		39 (40.6)	57 (59.4)	< 0.001	1.00 (Reference)	
Prophylaxis	21 (72.4)	8 (27.6)		4.22 (1.61–11.10)	0.003	48 (64.9)	26 (35.1)		2.37 (1.21–4.65)	0.012
Directed therapy	10 (38.5)	16 (61.5)		1.01 (0.39–2.59)	0.991	5 (12.2)	36 (87.8)		0.15 (0.05–0.52)	0.003
Reason for use documented, *n* (%)^d^										
Yes	39 (48.1)	42 (51.9)	0.838			88 (42.5)	119 (57.5)	0.035^b^	NA	NA
No	15 (44.1)	19 (55.9)				4 (100.0)	0 (0.0)		NA	NA
Antimicrobial group, *n* (%)										0.474
Penicillin	24 (40.7)	35 (59.3)	0.357^b^			16 (33.3)	32 (66.7)	0.029^b^	1.00 (Reference)	
Cephalosporin	13 (59.1)	9 (40.9)				40 (50.0)	40 (50.0)		1.50 (0.66–3.40)	0.335
Carbapenem	0 (0.0)	2 (100.0)				5 (27.8)	13 (72.2)		2.86 (0.65–12.70)	0.166
Nitroimidazole	3 (42.9)	4 (57.1)				18 (66.7)	9 (33.3)		2.38 (0.83–6.82)	0.108
Quinolone	5 (45.5)	6 (54.5)				2 (33.3)	4 (66.7)		1.01 (0.15–6.61)	0.995
Other	9 (64.3)	5 (35.7)				11 (34.4)	21 (65.6)		1.05 (0.38–2.90)	0.922

HCTM, Hospital Canselor Tuanku Muhriz; UMMC, University Malaya Medical Centre; OBGYN, obstetrics and gynecology.

^a^ Chi-Squared test.

^b^ Fisher Exact test. Odd ratio based on inappropriate group (inappropriate/appropriate).

^c^ *p*-value for Adjusted Odd Ratio.

^d^ Excluded in the multivariate analysis due to small number.

## 4 Discussion

This study constitutes a vital component of our ongoing AMS program, which utilizes PPS to delve into various facets of antimicrobial prescribing within our healthcare facilities. We seek to gain an understanding of these practices and to identify areas for enhancing the quality of care in surgical-practice units. This initiative represents an enduring commitment to fostering prudent antimicrobial usage and addressing the ever-pressing issue of antibiotic resistance.

### 4.1 Prevalence of antimicrobial prescribing in surgical-practice units

The overall usage of antimicrobials in our surgical-practice units at 44.2% was relatively lower compared to rates reported in African hospitals (Bediako-Bowan et al., 2019; Nnadozie et al., 2020), Asia (Limato et al., 2021; Sheng et al., 2019; de Guzman Betito et al., 2021), Italy (Macera et al., 2021), and Serbian hospitals (Šuljagić et al., 2021) (ranged 55.7%–97.6%). Conversely, other surveys, such as PPS in German (Aghdassi et al., 2018) and Belgian hospitals (Vandael et al., 2020), observed a lower prevalence of antimicrobial use, at approximately 30%. The variability in antimicrobial prescribing prevalence, both between our two hospitals, and in comparison to previous reports could be related to differences in the surgical-based case-mix, or structural characteristics unique to each hospital, including the type and proportion of surgical-based specialties. Moreover, significant differences were observed in the patterns of antimicrobial prescribing between the two hospitals, indicating the nature of using local guidelines, which provide various recommendations in accordance with each hospital policy, as well as considerations related to institutional antibiograms and costs, including administrative expenses. Our data also showed a higher usage of antibiotics classified as Watch antibiotics, particularly in UMMC. In response to the global concern of AMR, the AWaRe classification was developed as a general guide to antibiotic prescribing patterns aimed at promoting rational prescribing (World Health Organization, 2021). The WHO recommends at least 60% of all antibiotics prescribed nationwide to be from the Access group. Access antibiotics exhibit a wider range of activity against commonly susceptible pathogens, while sustaining lower resistance potential compared to antibiotics in the other groups. Watch group contain generally broader spectrum antibiotics, pose a higher risk of selecting antimicrobial resistance and are primarily used in patients with more severe conditions. Their use should be vigilantly monitored to prevent overuse. Integrating AWaRe index into our hospital policies shall be an essential measure, as it has been associated with improved usage of Access antibiotics (Budd et al., 2019), highlighting its potential benefits in promoting responsible antimicrobial use and combating AMR.

### 4.2 Compliance with guidelines and appropriateness

In this study, we identified appropriateness as the key measure of antimicrobial prescribing quality, moving beyond mere guideline compliance. This approach allowed us to consider various contexts in which non-compliance with the guidelines may not necessarily be deemed as inappropriate prescribing, but rather a case-specific approach that may still be adequately appropriate (Ierano et al., 2019a). However, it is important to note that due to variations in definitions of appropriateness and compliance across the literature, comparisons can be challenging and should be interpreted with caution. Ideally, the target for appropriate antimicrobial prescribing rates in surgical-based units should be above 90%, aligning with general goals for hospital-wide antimicrobial prescribing and SAP prescribing (Vandael et al., 2020; National Centre for Antimicrobial Stewardship and Australian Commission on Safety and Quality in Health Care, 2021). Alarmingly, our study revealed that both the rates of compliance with guidelines and appropriateness fell below this recommended threshold in the surveyed population. Only a small number of PPS studies reported the findings on surgical-practice units specifically, demonstrating compliance with guidelines that ranged from 70% to 92.7% (Elhajji et al., 2018; Singh et al., 2019; Vandael et al., 2020; Macera et al., 2021).

One of the main reasons for inappropriate prescribing in this study was the incorrect duration of antimicrobial prescriptions (15%), predominantly reflecting the extended use of SAP following surgery (41.3%). Best practice guidelines typically recommend a total SAP duration of less than 24 h for most procedures (Bratzler et al., 2013) and NAPS setting targets for this quality indicator at less than 5% (National Centre for Antimicrobial Stewardship and Australian Commission on Safety and Quality in Health Care, 2018). Unfortunately, there has been a persistent pattern of non-compliance and inappropriate prescribing for SAP documented in the literature (Kaya et al., 2016; Mousavi et al., 2017; Alemkere, 2018; Satti et al., 2019; Vicentini et al., 2019; Alahmadi et al., 2020; Bunduki et al., 2020; Khan et al., 2020; Prévost et al., 2020; Bardia et al., 2021; Cabral et al., 2023) ranging from a complete non-compliant to the national guideline (Alemkere, 2018) to 64% compliant (Prévost et al., 2020), while other reports varied from 40.9% inappropriate (Kaya et al., 2016) to 9.5% appropriate SAP use (Khan et al., 2020). Notably, Australian hospitals have monitored the key indicators of antimicrobial appropriateness using NAPS since 2013. While improvements have been observed in certain indicators (i.e., documentation), the proportion of SAP prescriptions exceeding 24 h has remained consistently high, at approximately 30% and has been static since 2015 (Australian Commission on Safety and Quality in Health, 2021). This persistence underscores the challenges of addressing this widespread issue. A comprehensive systematic review across various surgical subspecialties has also highlighted that extending prophylaxis duration does not confer additional reduction on the risk of surgical site infection when best practice (appropriate timing, dosage and re-dosing) is applied (de Jonge et al., 2020). In addition, prolonged SAP duration has been linked to increased risk of adverse events, including acute kidney injury and *Clostridiodes difficile* infection, contributing to the risk of acquired AMR (Harbarth et al., 2000; Bell et al., 2014; Bernatz et al., 2017; Branch-Elliman et al., 2019).

The proportion of SAP prescriptions in the study (27.1%) was higher compared to Australian Hospital NAPS reports for 2018 and 2019, ranging from 13.9% to 12.6% (National Centre for Antimicrobial Stewardship and Australian Commission on Safety and Quality in Health Care, 2021). Surveys conducted in Europe, Canada, Belgium and Thailand have reported a common preference of cefazolin for SAP (Versporten et al., 2018; Vandael et al., 2020; German et al., 2021; Anugulruengkitt et al., 2022). In contrast, our study observed a high usage of cefuroxime, which depicted similar therapeutic efficacy to cefazolin in preventing surgical site infections (Ahmed et al., 2022). This unconventional choice was influenced by local guidelines recommending cefuroxime, with or without metronidazole, and amoxicillin/clavulanic acid for most procedures due to the unavailability of cefazolin in our centers during the audit period, resulting in non-standard cefazolin use among prescribers. Similar antimicrobials were commonly employed in several lower-middle-income countries (LMIC) (Labi et al., 2018; Saleem et al., 2019; Umeokonkwo et al., 2019; de Guzman Betito et al., 2021), in accordance with their standard treatment guidelines (Bediako-Bowan et al., 2019). In line with global standards and recommendations, our recent guidelines have designated cefazolin as the first-line agent for the majority of procedures (Ministry of Health Malaysia, 2019; University Malaya Medical Center, 2020). While narrow-spectrum antimicrobial is recommended for SAP, inappropriate broad-spectrum antimicrobials were observed, with a dominance of third-generation cephalosporins (ceftriaxone and cefoperazone) and unnecessary anaerobe coverage with metronidazole. Ceftriaxone, a WHO Watch group antibiotic, is not recommended for SAP in our settings due to its lack of significant advantages over the first and second-generation cephalosporins, and its potential for resistance selection. Its usage is limited to cases of contamination or treatment for infection (Bratzler et al., 2013; Ministry of Health Malaysia, 2019). The preference for ceftriaxone in SAP can be attributed to its easy availability and long half-life, which eliminates the need for additional intra-operative doses. An extensive use of ceftriaxone as SAP in this study and various studies globally (Alemkere, 2018; Satti et al., 2019; Rachina et al., 2020; Limato et al., 2021; Fentie et al., 2022) ranging from 26.4% to 84%, poses another significant challenge for AMS.

Both hospitals also displayed a tendency to choose broader-spectrum coverage antimicrobials across all types of treatment (empiric, prophylaxis and directed therapy). In general, the antimicrobial sensitivity testing (AST) results serve as a valuable tool in determining the optimum antimicrobial therapeutic option, highlighting narrow-spectrum agents whenever possible and keeping in check broad-spectrum antimicrobials that exert higher selective pressure for AMR (Gajic et al., 2022). However, the accurate and timely AST performance is challenged by several factors in our hospitals. Proper interpretation of AST results with regard to efficacy and sensitivity among susceptible categories should be counselled by experts to provide individualized or personalized targeted treatment, as selecting antimicrobials based upon a direct comparison of susceptibility values obtained through *in vitro* testing could be misleading and inaccurate (Gajic et al., 2022). The absence or delay of laboratory data and AST in empiric therapy decisions often leads to the use of broad-spectrum antimicrobials, and at times, polypharmacy, inadvertently encouraging drug resistance (Chokshi et al., 2019).

In UMMC, a noteworthy pattern of non-compliance was identified, with significantly higher occurrence observed in general surgery, OBGYN and trauma and orthopedic units. Evidence of guideline compliance has yielded diverse outcomes in various prospective observational studies. NAPS reports on antimicrobial use in Canada indicated a commendable rate of appropriate prescription, notably in gynecology unit at 86.2% (CARSS, 2022). Conversely in Nigeria, an audit in OBGYN wards painted a different picture, highlighting excessive and inappropriate antimicrobial usage, with similar output including high incidence of redundant anaerobic coverage with metronidazole (Abubakar et al., 2018). Meanwhile, Thomas et al. (2022) found higher compliance in both gynecology (88.6%) and orthopedic (86.3%) compared to surgery (67.9%). Our study also highlighted that although documentation practices were significantly higher in UMMC, which utilizes electronic medical records (EMR) and electronic prescribing (e-prescribing), compared to HCTM, where paper-based health records are used, this criterion did not significantly influence the rates of compliance and appropriateness. However, King et al. (2017) and Hand et al. (2017) have outlined the potential of digital platforms and electronic health information technology in aiding prescribers throughout the antimicrobial lifecycle encompassing initiation, review, stopping and supplying of discharge medications. The technology is anticipated to have a positive impact on documentation and compliance in the surgical unit (Charani et al., 2017).

Despite these observations, the precise causes of the high non-compliant of prescribers in this study were uncertain and unexplored; thus, the explanation for this finding warrants further investigation. Insights drawn from an ethnographic study shed light on surgeons’ priorities, which primarily revolve around surgical procedures, surgical care and patient outcomes. Surgeons often place a strong emphasis on starting antimicrobials than on reviewing or stopping them, while rarely discussing the choice of antimicrobial (Charani et al., 2018). This potentially leading to prolonged and unnecessary use of these drugs. A review by Hassan et al. identified a common barrier to compliance with guidelines stemming from prescribers’ inadequate knowledge and unfamiliarity of guideline content (Hassan et al., 2021). However, Ierano et al. (2019b) highlighted that prescriber preferences and autonomy are often considered more important than strict compliance with guidelines, even when prescribers are well-informed about the guidelines. Moreover, guidelines are often viewed as general recommendations that lack the necessary details to address the diverse array of surgical procedures and various patient characteristics and environmental factors that complicate decision-making in complex situations. A recent survey conducted among Surgical Infection Society (SIS) members, experts in surgical infections, revealed that surgeons hold varying opinions regarding the appropriate duration of prophylaxis and therapeutic antimicrobials for inpatients across common indications (Delaplain et al., 2022). It is evident that heightened prescribers’ awareness regarding their prescribing practices is a crucial component of AMS efforts.

## 5 Limitations

While the PPS is capable of presenting the overview of antimicrobial usage in surgical-practice units, we believe the widespread use of the extended duration of antimicrobial post-surgery is underestimated, given that the survey methodology does not capture the intricacy of preoperative, intraoperative and post-operative antimicrobial use. Second, the results from two tertiary teaching hospitals may not be generalized to all surgical-practice units across hospitals in the country but still they are required to set direction and targets for AMS interventions. It is also an important contribution to drive a change in prescribing and policy development. Third, a variable degree in assessment is possible, as interpretations may differ from one another. However, an assessment tool and support from the Australian NAPS were available to assist with any disagreement throughout the study. Another limitation is the absence of quantitative measures such as defined daily doses (DDD) to quantify antimicrobial consumption, as this study focused primarily on qualitative assessment of antimicrobial practices. Future study may benefit from incorporating quantitative measures to complement qualitative assessment of antimicrobial prescribing practices.

## 6 Conclusion

This study provides valuable insights into the antimicrobial usage, indications and determinants of non-compliance and inappropriateness within the surgical-practice units of two teaching hospitals in Malaysia. The findings emphasized the urgent need for a strong commitment of AMS initiatives that focus on reducing unnecessary prolongation of SAP and unnecessary use of broad-spectrum antimicrobials to enhance rational prescribing in the surgical field. It is recommended that the WHO AWaRe classification be incorporated into the national and local antimicrobial guidelines, as well as embedded in the AMS quality improvement program to facilitate monitoring and restriction of Watch antibiotics, which carry higher risk of resistance potential. A collective work by actively involving and raising awareness among prescribers is crucial to promote proper documentation, encouraging guidelines compliance and favoring overall appropriateness to ensure responsible use of antimicrobial in surgical settings.

## Data Availability

The original contributions presented in the study are included in the article/Supplementary Material, further inquiries can be directed to the corresponding author.
