# Supplementary material for: Assessment of antimicrobial prescribing patterns, guidelines compliance, and appropriateness of antimicrobial prescribing in surgical-practice units: point prevalence survey in Malaysian teaching hospitals
Source: Front Pharmacol. 2024 Apr 24;15:1381843. doi: 10.3389/fphar.2024.1381843 (PMC11076853; doi:10.3389/fphar.2024.1381843)
Supplement: Supplementary file 1 [file Table1.docx]

Table S1 summarises the AWaRe categories and prescribing patterns in both hospitals

| AWaRe categories |  | Total | HCTM, n (%) | UMMC, n (%) | P value* |
| --- | --- | --- | --- | --- | --- |
| ***Access*** |  | ***133*** | ***59*** | ***74*** | *0.017* |
| Indicates the first or second choice antibiotics for most common infections.  These antibiotics offer the best therapeutic value, should be available at all times while minimizing the potential for resistance | *Empiric* | *74 (55.6)* | *30 (50.8)* | *44 (59.5)* |  |
|  | *Prophylaxis* | *42 (31.6)* | *16 (27.1)* | *26 (35.1)* |  |
|  | *Definitive* | *17 (12.8)* | *13 (22.0)* | *4 (5.4)* |  |
|  |  |  |  |  |  |
| ***Watch*** |  | ***196*** | ***55*** | ***141*** | *0.087* |
| Indicate the first or second choice antibiotics but only indicated for specific, limited number of infective syndromes.  These antibiotics more prone to be a target of antibiotic resistance and thus prioritized as targets of stewardship programs and monitoring. | *Empiric* | *86 (43.9)* | *31 (56.4)* | *55 (39.0)* |  |
|  | *Prophylaxis* | *62 (31.6)* | *13 (23.6)* | *49 (34.8)* |  |
|  | *Definitive* | *48 (24.5)* | *11 (20.0)* | *37 (26.2)* |  |
|  |  |  |  |  |  |
| ***Reserve*** |  | ***1*** | ***1*** | ***0*** | *NA* |
| Antibiotic that should be only used as a “last resort” when all other antibiotics have failed. Their use should be tailored to highly specific patients (life-threatening infections due to multi-drug resistant bacteria)  These antibiotics could be protected and prioritized as key targets of stewardship programs to ensure their continued effectiveness | *Definitive* | *1 (100)* | *1 (100)* | *0* |  |
|  |  |  |  |  |  |
| ***Unclassified*** |  | ***9*** | ***4*** | ***5*** | *0.444^* |
| *Empiric* |  | *8 (88.9)* | *3 (75)* | *5 (100)* |  |
| *Definitive* |  | *1 (11.1)* | *1 (25)* | *0* |  |

**Chi-Squared test, ^Fisher Exact test.*

Resource: World Health Organization, “Access, Watch, Reserve, classification of antibiotics for evaluation and monitoring of use,” Geneva, 2021.
