# Supplementary material for: Assessment of antimicrobial prescribing patterns, guidelines compliance, and appropriateness of antimicrobial prescribing in surgical-practice units: point prevalence survey in Malaysian teaching hospitals
Source: Front Pharmacol. 2024 Apr 24;15:1381843. doi: 10.3389/fphar.2024.1381843 (PMC11076853; doi:10.3389/fphar.2024.1381843)
Supplement: Supplementary file 2 [file Image2.pdf]

HOSPITAL NAPS National Antimicrobial Prescribing Survey

Hospital NAPS appropriateness definitions

GUIDANCE | NCAS

			If endorsed guidelines are present	If endorsed guidelines are absent
Appropriate	1	**Optimal**[1]	Antimicrobial prescription follows either the Therapeutic Guidelines[2] or endorsed local guidelines *optimally*, including antimicrobial choice, dosage, route and duration[3]	The antimicrobial prescription has been reviewed and endorsed by an infectious diseases clinician or a clinical microbiologist<br>**OR**<br>The prescribed antimicrobial will cover the likely causative or cultured pathogens ***and*** there is not a narrower spectrum or more appropriate antimicrobial choice, dosage, route or duration[3] available
	2	**Adequate**	Antimicrobial prescription does not optimally follow the Therapeutic Guidelines[2] or endorsed local guidelines, including antimicrobial choice, dosage, route or duration[3], however, is a ***reasonable*** alternative choice for the likely causative or cultured pathogens<br>**OR**<br>For surgical prophylaxis, as above ***and*** duration[3] is less than 24 hours	Antimicrobial prescription including antimicrobial choice, dosage, route and duration[3] is not the most optimal, however, is a ***reasonable*** alternative choice for the likely causative or cultured pathogens<br>**OR**<br>For surgical prophylaxis, as above ***and*** duration[3] is less than 24 hours
Inappropriate	3	**Suboptimal**	There may be a mild or non-life-threatening allergy mismatch<br>**OR**<br>Antimicrobial prescription including antimicrobial choice, dosage, route and duration[3], is an ***unreasonable*** choice for the likely causative or cultured pathogens, including:<br>• spectrum excessively broad, unnecessary overlap in spectrum of activity, dosage excessively high or duration excessively long<br>• failure to appropriately de-escalate with microbiological results	
	4	**Inadequate**	Antimicrobial prescription including antimicrobial choice, dosage, route or duration[3] is ***unlikely*** to treat the likely causative or cultured pathogens<br>**OR**<br>The documented or presumed indication does not require ***any*** antimicrobial treatment<br>**OR**<br>There may be a severe or possibly life-threatening allergy mismatch, or the potential risk of toxicity due to drug interaction<br>**OR**<br>For surgical prophylaxis, the duration[3] is greater than 24 hours (except where local guidelines endorse this)	
	5	**Not assessable**	The indication is not documented and unable to be determined from the notes<br>**OR**<br>The notes are not comprehensive enough to assess appropriateness<br>**OR**<br>The patient is too complex, due to multiple co-morbidities, allergies or microbiology results, *etc.*	

[1] Taking into account acceptable changes due to the patient's weight, allergy status, renal or hepatic function, or relevant drug interactions (if this information is available)

[2] Antibiotic Expert Group. Therapeutic Guidelines: Antibiotic. Version 16 (2019), or online version

[3] Duration should only be assessed if the guidelines state a recommended duration and the antimicrobial has already been dispensed for longer than this, or if there is a clear planned 'end date' documented

Doc:nNAPS.AD.v6.1; 20190513

Resource: https://www.naps.org.au/Resources/Hospital_NAPS-ADv6.1_20190513.pdf
