# Supplementary material for: Assessment of antimicrobial prescribing patterns, guidelines compliance, and appropriateness of antimicrobial prescribing in surgical-practice units: point prevalence survey in Malaysian teaching hospitals
Source: Front Pharmacol. 2024 Apr 24;15:1381843. doi: 10.3389/fphar.2024.1381843 (PMC11076853; doi:10.3389/fphar.2024.1381843)
Supplement: Supplementary file 3 [file Image1.pdf]

S1 Data collection form

HOSPITAL NAPS National Antimicrobial Prescribing Survey

GUIDANCE | NCAS

Audit date	Patient identification number	Date of birth / age	Gender	Specialty ❐ *currently in ICU / NICU*	Ward	Weight *kg*	eGFR / CrCl *ml/min*
/ /		/ /	M / F / O				

Antimicrobials

Only record the antimicrobials as prescribed at 08:00 am on the audit day and any surgical prophylaxis or stat doses in the previous 24 hours

For NICU patients Birth weight *kg*	Gestational age *weeks*

Start date	Antimicrobial	Route	Dose	Freq	Indication documented	Specify documented or presumed indication	Review / stop date documented	Guideline compliance (1-6)	Surgical prophylaxis > 24 hrs	Allergy mismatch	Microbiology mismatch	Incorrect route	Incorrect dose / frequency	Incorrect duration	Spectrum too broad	Spectrum too narrow	Indication does not require any antimicrobials	If restricted: approval given	Appropriateness (1-5)
/ /																			
/ /																			
/ /																			
/ /																			
/ /																			

Allergies and adverse drug reactions to antimicrobials

❐ nil known ❐ not documented

❐ present; *record the antimicrobial and the nature of the reaction*

Microbiology ❐ not collected / not assessable

❐ collected; *record the specimen type, organism and susceptibilities if relevant*

Guideline compliance

1. Compliant with Therapeutic Guidelines
2. Compliant with locally endorsed guidelines*
3. Non-compliant with guidelines
4. Directed therapy
5. No guidelines available
6. Not assessable

*Select **Therapeutic Guidelines** if local guidelines are the same

Clinical notes or comments

❐ Renal replacement therapy given within the previous 24 hours; *eg. dialysis*

Surgical procedure if performed

If prophylaxis given within previous 24 hours; *include in audit*

Appropriateness

1. Optimal
2. Adequate
3. Suboptimal
4. Inadequate
5. Not assessable

Doc:HospitalNAPS-DCFv7;20160705

Hospital-wide point prevalence survey. This methodology required all inpatients to be assessed so prevalence of antimicrobial use could be calculated. Data were collected on both the number of inpatients on antimicrobials (numerator) and the total number of inpatients (denominator). This option is recommended to be completed on a single calendar day. However, if this was not possible, wards could be surveyed on separate days provided that all patients were surveyed once only. Source: James et al. The feasibility and generalizability of assessing the appropriateness of antimicrobial prescribing in hospitals: a review of the Australian National Antimicrobial Prescribing Survey. Vol. 4, JAC-Antimicrobial Resistance. 2022.
